# Supplementary material for: Phenol Degradation by Pseudarthrobacter phenanthrenivorans Sphe3
Source: Microorganisms. 2023 Feb 18;11(2):524. doi: 10.3390/microorganisms11020524 (PMC9966258; doi:10.3390/microorganisms11020524)
Supplement: Supplementary file 1 [file microorganisms-11-00524-s001.zip › microorganisms-2218415-supplementary.pdf]

Supplementary Materials

Table S1. Oligonucleotides used for the RT-qPCR experiments.

Gene Locus Tag/ Gene Name	Oligonucleotides Designation	Oligonucleotides Sequence (5'→3')	Size (bp)
Asphe3_36590/*phehydrox*	phehydroxfor	GTCACCGACTTCCCCGATAT	167
	phehydroxrev	ATCTGCTCGATGGTGGTGTT	
Asphe3_35170/*cat12diox*	cat12dioxfor	AAACGGATACCCGGAAAGAG	198
	cat12dioxrev	GGGCGTTGTACTCCTCGTAG	
Asphe3_40510/*cat23diox*	cat23dioxfor	AGCCAGTTCCACCACGATAT	182
	cat23dioxrev	CAATACTGGTTTCCGCCGAC	
Asphe3_00060/*gyrβ*	gyrβfor	GGCTAACGACAATACAGATA	210
	gyrβrev	ACCACTTCATAAACAAGGT	

Table S2. Measurements of remaining phenol in Sphe3 cultures in various phenol concentrations, at selected timepoints.

Time (Hours)	MEAN (N=3)	SD (±)
	**300 mg/L**	
0	301,3	3,2
6	245,3	1,5
12	244,3	1,5
24	243,6	4,5
	**500 mg/L**	
0	500,3	2,5
6	348,6	1,2
12	345	3
24	345,6	2,5
	**750 mg/L**	
0	748,6	2,3
6	448,6	4,1
12	448,3	1,5

24	445	1
	**1000 mg/L**	
0	1002,3	2,5
6	671,6	1,5
12	610,6	10,1
24	499,6667	10
	**1200 mg/L**	
0	1200	3
6	869	6,1
12	829,6667	2,5
24	819	3,6
	**1500 mg/L**	
0	1503	2,6
6	1043,3	7,6
12	1043,3	15,3
24	1021,6	12,6

Table presents means of three measurements (N=3), along with the standard deviations (±SD**)**.

Table S3. BLASTP search of Sphe3 phenol hydroxylase and catechol dioxygenases.

**Sphe3 Gene Under Study**	**Enzyme Description**	**Microorganism**	**Query Coverage (%)**	**Per. Identity (%)**	**Length (aa)**	**Accession No.**
Asphe3_36590	phenol 2-monooxygenase	*Arthrobacter* sp. NtRootA9	99	87.12	635	BCW20813.1
		Arthrobacter sp. OY3WO11	100	87.42	636	OAE03256.1
		Arthrobacter sp. PvP023	98	86.51	638	MBP1134756.1
		Arthrobacter sp. Leaf137	99	85.90	632	KQQ83477.1
		Arthrobacter sp. OV608	100	86.79	636	SER24611.1

		Arthrobacter sp. OV608	100	84.80	638	SER22274.1
		Arthrobacter sp. StoSoilB22	99	84.29	644	BCW63098.1
Asphe3_35170	Catechol 1,2-dioxygenase	*Arthrobacter* sp. BB-1	100	92.62	298	TNB68529.1
		Arthrobacter sp. SPG23	100	92.28	298	WP_043481025.1
		Arthrobacter sp. PAMC25564	100	91.61	298	WP_136321068.1
		Pseudarthrobacter sp. GA104	100	91.95	298	MUU73784.1
Asphe3_40510	Catechol 2,3-dioxygenase	*Rhodococcus wratislaviensis* IFP 2016	100	56.46	293	ELB89778.1
		Geobacillus genomosp. 3	100	46.26	296	BAD08308.1
		Hydrogenibacillus schlegelii	100	45.64	298	PTQ51344.1
		Pseudaminobacter salicylatoxidans	100	38.61	301	PWJ76351.1

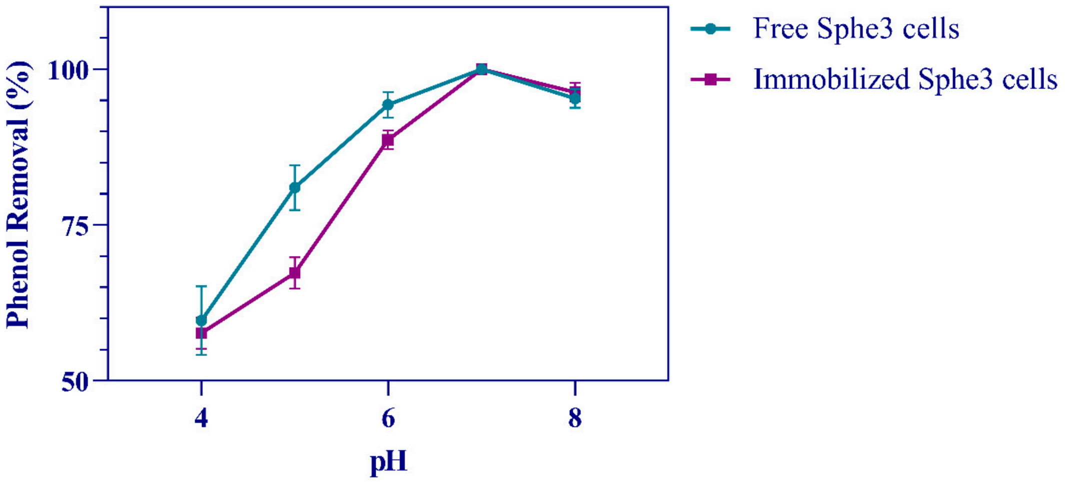

Figure S1. Effect of pH on the phenol removal activity of Sphe3 in its free form (circle) and after immobilization (square). The activities at the optimal pH were set to 100% for each case.

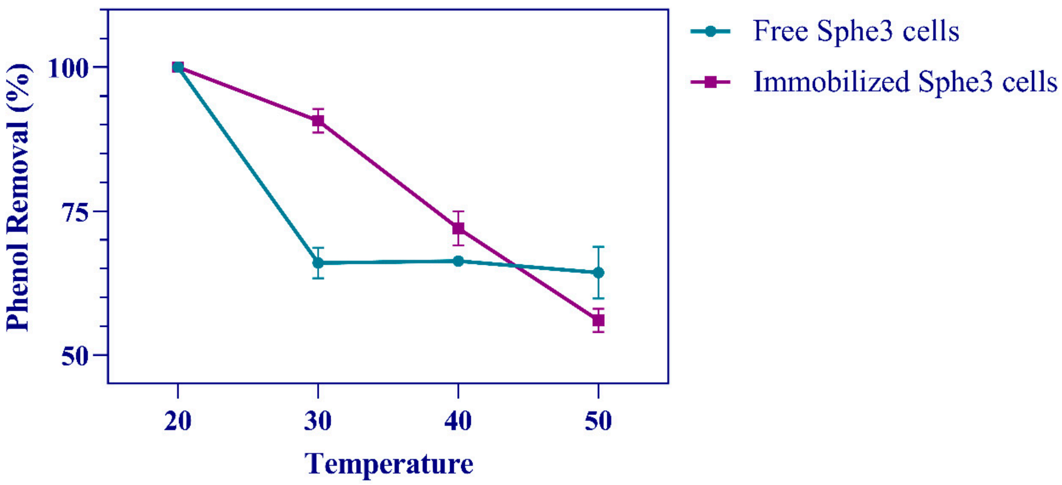

Figure S2. Effect of temperature on the phenol removal activity of Sphe3 in its free form (circle) and after immobilization (square). The activities at the optimal temperature were set to 100% for each case.
